# Do All Patients Require Radiotherapy after Breast-Conserving Surgery?

**DOI:** 10.3390/cancers2020740

**Published:** 2010-04-28

**Authors:** Anita R. Skandarajah, G. Bruce Mann

**Affiliations:** 1 Department of Surgery, The University of Melbourne, Parkville, 3050, Australia; 2 The Breast Service, The Royal Melbourne and Royal Women’s Hospital, Parkville 3050, Australia; E-Mail: Bruce.Mann@mh.org.au

**Keywords:** breast conservative therapy, radiotherapy, ipsilateral breast tumor recurrence

## Abstract

Radiotherapy following breast conservation is routine in the treatment of breast cancer. This creates a large demand for radiotherapy services with implicit cost effects and potential morbidity to patients. Radiotherapy is administered to decrease local recurrence, but is radiotherapy required for all breast cancers? A literature search using the Medline and Ovid databases was conducted between 1965 and 2010 using the terms ‘role of radiotherapy’, ‘early breast cancer’, and omission of radiotherapy’. Papers with clinical trials published in English in adult humans were included. Fourteen randomized controlled trials were included. Local recurrence rates range from 0.8–35% in patients in whom radiotherapy was omitted. Low risk characteristics include older age, small tumor size, no lymphovascular invasion and low to moderate grade. At present, there is no clearly defined low risk group of patients in whom radiotherapy can be omitted.

## 1. Introduction

Until the 1970s, local management of breast cancer was total mastectomy and axillary dissection. Multi-center randomized controlled trials such as NSABP B-06 and the MILAN trial with 20 years of follow-up comparing total mastectomy with wide local excision and adjuvant whole breast radiotherapy, then demonstrated that there is no significant difference in overall survival [1,2]. As a result, standard practice in the management of early and small breast cancers has become breast-conserving therapy (BCT) - wide local excision with negative margins and radiotherapy (RT).

Radiotherapy is offered almost routinely as part of BCT in early breast cancer to decrease local recurrence, as groups that can be safely treated with wide excision alone have not been clearly identified based on clinical and pathological features of the excised cancer. This has resulted in a large demand for radiation therapy, with substantial inconvenience, short-term and potentially long-term side effects and a cost to the community of at least $8,500 (USD) per patient. Limited access to radiotherapy for patients in regional and remote areas can result in patients choosing total mastectomy to avoid radiotherapy [3,4,5].

The main goal of radiotherapy is to decrease local recurrence. A local recurrence occurs in the same quadrant of the ipsilateral breast. In this systematic review, we examine the evidence for omission of radiotherapy in early breast cancer and attempt to identify a group at low risk of local recurrence based on clinicopathological risk factors.

## 2. Methods

A literature search using the Medline and Ovid databases was conducted between 1965 and 2010 using the terms ‘role of radiotherapy’, ‘early breast cancer’, omission of radiotherapy’ and a combination of these terms. Only papers with clinical trials published in English in adult humans with available abstracts were included. Papers regarding partial breast irradiation were excluded. The “related articles” function was used to broaden the search, and all abstracts, studies, and citations scanned were reviewed. The references from articles were also used. All the papers included were cases series with a level of evidence of three as a minimum requirement. The evidence was critically examined. 

## 3. Results

There are 14 published randomized controlled trials evaluating the omission of radiotherapy in early breast cancer and one meta-analysis, published at two five-year intervals.

### 3.1. Meta-Analyses

The Oxford overview published in 2000 [6], evaluated the role of radiotherapy in 19582 women, half of whom were node positive. Radiotherapy fields used in these trials were not as targeted as those used today, and often routinely included chest wall, axillary, supraclavicular and internal mammary nodes. A reduction in local recurrence was seen in all patients receiving RT with a LR rate of 8.8% *versus* 27.2% at 10 years, with a small improvement in survival in women with high risk tumors, particularly women of young age and node positive tumors. Patients receiving adjuvant endocrine therapy were under-represented in these trials and thus do not mirror modern practice. The Oxford overview also highlighted that radiotherapy increased the deaths unrelated to breast cancer by 21% [6], in particular vascular mortality subsequent to inadvertent coronary irradiation. However, modern radiotherapy techniques minimize coronary and pulmonary exposure thus causing negligible risk [7]. Low risk groups particularly underrepresented in this overview were women with estrogen receptor (ER) positive tumors receiving adjuvant hormonal treatment (70% of breast cancers are ER positive) and women over the age of 70. The overview authors concluded that any benefit in local recurrence and breast-cancer specific mortality must be weighed against the risks of radiotherapy especially for those at low risk of recurrence (as defined by screen-detected, node negative and elderly patients in this overview). Although this is a highly powered study, application to today’s population is difficult as the majority of women present as screen-detected early breast cancers. Improvements in both radiotherapy techniques and use of adjuvant hormonal therapy also affect the studies applicability.

An update of the Oxford overview was published in 2005, and analyzed 23,500 patients including 7,300 patients who had breast-conserving surgery. An overall risk reduction in local recurrence of 19% from 26% to 7% (29% to 10% in node-negative women) and 5% reduction in both breast-cancer specific (36% to 31%) and overall mortality [8]. Across all groups, a reduction in four local recurrences was associated with one fewer breast cancer death at 15 years. In low risk groups, defined by a <10% difference in local recurrence, in those with and without RT, a smaller benefit in overall mortality was seen. Once again, the authors concluded that the older radiotherapy regimens resulted in a significant number of adverse events including increased cardiac and lung cancer mortality and contralateral breast cancer (rate ratio 1.8, 2p = 0.002). An analysis to define low risk groups identified node-positivity as a major risk for local recurrence and in the node-negative group, young age, poor tumor differentiation and large tumor size were associated with increased local recurrence.

This overview was once again criticized for the under-representation women over 70, for including large tumors and the inclusion of cases where the axillary status was unknown [8]. The authors duly acknowledged these limitations and recognized that screening has enabled earlier diagnosis, that adjuvant therapy is now more prevalent and that the risk of local recurrence in these groups will be much lower. The changes in radiotherapy technology has also resulted in safer breast treatment, reducing the number of adverse events from radiotherapy The conclusion was that the local recurrence and survival benefits must be counter-balanced with the cosmetic and functional effects of local treatment.

### 3.2. Randomized Controlled Trials

The randomized controlled trials of the 1980s reported local recurrence rates at five years of 18–39% [1,2,9,10] in those who did not receive RT. These trials also included all tumor sizes up to 5 cm and some included lymph node positive patients. Fisher *et al.* published the 20 year follow-up results of NSABP B-06 which included tumors up to size of 4 cm [2]. This trial evaluated the difference between mastectomy *versus* lumpectomy with or without radiation. A 19% (36% *versus* 17%) difference in local recurrence was seen in node negative patients receiving radiotherapy and 35% (44% *versus* 9%) in all groups. The difference in overall survival was not significant. The authors also acknowledged that current adjuvant therapy is more effective in decreasing local recurrence compared to regimens used in this trial.

Veronesi *et al*. published 10 year results of their randomized trial which also showed a benefit of 16% (21% *versus* 5%) for those receiving radiotherapy and identified younger age (<55) and node positivity as higher risk groups that clearly benefited from radiotherapy [11]. Patients over 65 had no significant benefit from radiotherapy. Clark *et al*. [12] have shown a similar decrease in local recurrence but with no overall survival benefit identifying a high risk group of age < 50, size > 2 cm and high nuclear grade. Renton *et al*., whose study of 418 patients did not mandate microscopically clear margins, identified margin positivity as the highest predictor of local recurrence [13]. In the Scottish Breast Cancer Trials Group study, 585 patients under age 70 with tumors <4 cm were included with a 1 cm tumor resection margin. Patients received adjuvant Tamoxifen or CMF dependent on their receptor status and were then randomzed to post-operative radiotherapy or no further treatment. Local recurrence was also decreased with radiotherapy (26% *versus* 6%) irrespective of ER status and adjuvant Tamoxifen.

In summary, all randomized trials of the 1980s showed a significant decrease in local recurrence, but no change in overall survival for those receiving radiotherapy (Table 1). These trials variably included large tumors up to 5 cm, all tumor grades, varying age, variable excision margins, tumors with differing hormone-sensitivity and nodal status. Variable excision margins definitions, for example, distance from tumor margins *versus* ‘no tumor at inked margin’ and non-standardization of pathology reporting are also potential confounding factors. However, they consistently found that involved margins, nodal positivity and young age were associated with a higher risk of local recurrence.

Defining low risk groups in older studies has been a challenge in planning Phase II and Phase III studies exploring omission of radiotherapy [14,15,16]. Seven more recent randomized trials have attempted to identify a group of early breast cancers where the benefits of radiotherapy may be insignificant. The CALGB trial C9343 included women over 70, with ER positive tumors < 2 cm, who were clinically node-negative and received hormone therapy. They showed a LR rate of 4% in those with no radiotherapy *versus* 1% in those receiving it at a median follow-up of five years. An update at 7.9 years showed a 5% benefit (6.3% *versus* 1% with RT), and identical breast-cancer specific mortality (2%) and overall all-causes mortality of 26% [17]. The major limitation of this study is the lack of pathological confirmation of axillary node status (64% did not have axillary assessment). The inclusion of pathologically node positive women may have increased the loco-regional recurrence rate. Despite the small difference in local recurrence, the authors concluded that, given no difference in regional or distal metastases, these low risk women could reasonably be treated with adjuvant tamoxifen alone [18].

Similarly, Fyles *et al*. in a study of 769 patients over age 50, included both T1 and T2 tumors. The group was evenly randomized to receive Tamoxifen and breast irradiation *versus* Tamoxifen alone. Stratification was according to tumor size (T1 *versus* T2), ER status, and center. A 6% benefit was seen with RT with 4.6% benefit in local recurrence in the T1 subset (3.2% *versus* 7.8%) [19]. A university analysis identified the following adverse prognostic features: tumors size > 2 cm, estrogen-receptor negativity, omission of radiotherapy and a higher pathological grade. An exploratory subgroup analysis concluded that patients with T1 tumors, who were > 60 and who were not receptor negative may be a low risk group. The authors concluded that in women greater than 70, who had early ER positive tumors, the addition of radiotherapy did not decrease mastectomy rate for local recurrence (1% in the irradiated group *versus* 2%) or increase the disease-free (99% *versus* 98%) or overall survival rate (87% *versus* 86%). However, the number of events in the population was small and the authors concluded appropriately that the lack of benefit of radiotherapy may be due inadequate power.

**Table 1:** Summary of randomized controlled trials evaluating omission of radiotherapy in early breast cancer.

Author/Group	Trial period	Patients	Age	Tumor	Grade	% node	Surgery	Margins	Systemic therapy	Follow-up	Local recurrence	
			restrictions	Size cm		positive					BCS	BCS + RT
**Randomized controlled trials**												
Fisher *et al.*	1976–1984	1137	None	≤4	All	35.4	WLE	R0	N1 only	20 years	27.9	7.7
*NSABP B-06[2]*											23.5 (N0)	10.4 (N0)
Liljegren *et al.*	1981–1988	381	None	≤2	All	0	Quad	R0	None	5 years	18.4	2.3
*Uppsala-Orebro[20]*												
Veronesi *et al.*	1987–1989	579	None	≤2.5	All	30.3	Quad	R0	N1 post-menopausal	10 years	23.5	5.8
*Milan III[11]*									received TAM			
Clark *et al.*	1984–1989	837	None	≤4	All	None	WLE	R0	None	43 months	25.7	5.5
*Ontario[12]*								(0.5–1 cm)		Median		
Renton *et al.*	1981–1990	418	None	≤5	All		WLE	Included	Yes if node positive or ER-ve		35	13
*British[13]*								incomplete		5 years		
Forrest *et al.*	1985–1991	585	<70	≤4	All	22.9	WLE	1 cm	All if ER + ve	5.7 median	24.5	5.8
*Scottish CTBG[21]*												
Hughes *et al.*	1994–1999	636	>70	≤2	All	None	WLE	R0	TAM 5 years	5 years median	4	1
*CALBG*												
CALBG update C9343[18]	1994–1999	636	>70	≤2	All				TAM 5 years	7–9	6.3	1
Spooner *et al.*	1995–	707		≤4	All	None	WLE	R0		2 years	13	4
*West Midland[22]s*												
Fyles *et al.*	1992–2000	769	None	≤5	All	None	WLE	R0	TAM 5 years	5.6 years	7.7	0.6
*Canadian[19]*	Subgroup			≤2						Median	5.9	0.4
*NSABP B-21[23]*	1989–1998	1009	None	≤1	All		WLE	>1 cm	TAM 5 years	8 years	16.5	2.8
BASO II[24]*	2004–	1062	None	≤2	All	None	WLE	R0	TAM 5 years	4 years	5	2
PRIME II[15]*	2004–	>240	>65	3	I,II	None	WLE	R0	All if ER + ve for 5 years	5 years		
*Holli et al[25].*	1990–1999	264	>40	≤2	I, II	None	WLE	1 cm	None	12 years	27	12
*Tinterri et al[26].*	2001–2005	749	55–75	<2.5 cm	NS	16 v 14	WLE	R0	Yes if node positive	53 months	2.7	0.7
*Winzer et al.[27]*	1991–1998	347	45–75	≤2	I,II	None	WLE	R0	Randomized to TAM	9.9 years	34	0.09
										With TAM	0.08	0.07

* interim results published in abstract form only.

The most compelling data supporting routine adjuvant radiotherapy after complete local excision comes from the NSABP B21 trial which included 1009 tumors in a three-armed study of Tamoxifen alone, RT alone or Tamoxifen and RT. Tumor size <1 cm and patients were treated with adjuvant Tamoxifen; an 11% decrease with radiotherapy was demonstrated. [23]. Local recurrence was similar across all ages. The authors concluded that radiotherapy should still be routine in these low risk women. They observed that 38% of the local recurrences occurred after five years and suggested that the follow-up may be too short in studies supporting omission of radiotherapy. This report like many others did not differentiate between a local recurrence and an ipsilateral new primary cancer. Although NSABP B21 did not include node positive or nodal status unknown patients (Nx) the authors did not report other higher risk factors such as lymphovascular invasion (LVI) or associated DCIS.

More recently, Holli *et al*. [25] published a study of 264 patients over age 40 with ≤20 mm tumors and a tumor-free margin of greater than 1 cm. All patient had nodal assessment by axillary dissection and were included if node negative, progesterone receptor positive, moderately to well-differentiated, low cell proliferation rate and unifocal. The local recurrence rate was 27% *versus* 12% in those receiving radiotherapy. However, no patient received adjuvant endocrine therapy. Tinterri *et al*. [26] have recently published their prospective randomized trial in an elderly age group (55–75 years) of 749 patients. Patients with tumors <2.5 cm with R0 resections and <3 nodes positive were included regardless of receptor status (88% receptor positive). Systemic treatment was given according to nodal status and biological need and adjuvant endocrine therapy in receptor positive disease. This study closely mirrors current standard treatment of breast cancer in this decade. At a minimum of 53 months follow-up, they had a recurrence rate of 2.5% in the surgery alone group and 0.7% in those who received radiotherapy with no difference in distant disease free survival or overall survival.

Interim results of the BASO II trial have been published in abstract form. This trial involved a clearly defined group of 1062 patients, with tumor size <2 cm who have had five years of adjuvant tamoxifen (ER positive) , who were randomized to receive adjuvant whole breast radiotherapy *versus* none [24]. Local recurrence occurred in 0.6% *versus* 0.8% receiving radiotherapy. The unpublished PRIME II study [15] has reached accrual targets and follow-up is ongoing. The study includes women >65 years, with ER positive, node-negative tumors < 3 cm in size, who undergo breast-conserving surgery and receive adjuvant endocrine treatment [28]. Patients with Grade III and patients with LVI were excluded. In their prior quality of life study (PRIME I) with the same cohort of patients, they had not detected any local recurrences at 15 months [29].

The most recent published randomized trial by Winzer *et al*. involves four arms randomizing low risk patients (<2 cm) to radiotherapy or not and tamoxifen use or not [27]. This study, albeit underpowered, has shown a benefit with both radiotherapy and tamoxifen reducing LR (recurrence rate of 34% in BCT alone) but of particular interest, is the group receiving tamoxifen with or without radiotherapy and the difference in LR of 0.08% to 0.07%. No significant difference could be established between the four treatment groups for distant disease-free survival rates.

### 3.3. Non-Randomized Studies

In a prospective study evaluating omission of RT in T1 node negative cancers, without extensive intraductal component and LVI, Lim *et al*. showed a recurrence rate of 23% (annual 3.5 per 100-patients years of follow-up) and concluded that the elderly may be the only group where radiotherapy omission may be considered [30]. Truong *et al.* included cancers up to 5 cm in their large retrospective series of 4,836 patients who did not receive adjuvant radiotherapy after BCS, and observed that not only were elderly patients less likely to receive radiotherapy and less likely to be included in trials, but that the overall survival of those elderly patients who did not receive RT was less than those who did. However, the subgroup analysis showed a non-significant difference in breast-cancer specific mortality except for the group >75 years. Factors influencing breast-cancer specific survival in their multi-variate analysis were not age, but lymphovascular invasion, node positivity, tumour size and radiotherapy. One limitation acknowledged in the study is the lack of data on comorbidities and the impact they may have on treatment recommendations as well as breast-cancer specific and overall survival.

In 2006, Lim *et al.* published an update of their prospective study omitting RT in Stage 1 breast cancer with low risk and found an IBTR of 23%. The authors concluded that apart from the elderly population with co-morbidities, radiotherapy should be routinely recommended [30]. However, no patient received adjuvant systemic therapy. In comparison, Varghese *et al.* have recently published a series of 173 patients <1 cm tumors and a tumor free margin and demonstrated a recurrence rate of 5% *vs.* with 6% (no RT). The lack of significant difference in local recurrence rates was in a setting of incomplete adjuvant hormonal administration and inclusion of patients with LVI [31].

### 3.4. What Factors Can Be Considered Low Risk?

The evidence supporting post-operative radiotherapy following breast-conserving surgery for invasive cancer is compelling and its impact on subsequent survival has been demonstrated by the meta-analyses and highlights the importance of local control. However, radiotherapy comes at a considerable cost with potential cosmetic and quality of life implications to the patient. The major task therefore is to define this low risk group.

The previous trials have been variable in identifying clinico-pathological factors which confer low local recurrence risk. These include older age, especially >70, small tumor size, non-high grade, clear resection margins, hormone receptor positivity and node negativity. Other factors known to be associated with low risk for local recurrence include no lymphovascular invasion, minimal or no DCIS and no multifocality and age [32,33,34]. Although some clinico-pathological factors can be determined pre-operatively on core biopsy, extent of DCIS and LVI are better assessed on the final pathological specimen. Intraductal carcinoma may be clearly identified with mammography if calcified, but is more difficult in non-calcified cases.

In one retrospective review of 2,243 patients with ipsilateral breast recurrence (IBTR), Yoshida *et al*. [35] found that margin (R0 *versus* R1) and omission of radiotherapy were significant on multivariate analysis for local recurrence. Age, PR positivity, LVI and IBTR but not RT per se were risk factors for disease free survival. Biologically it remains unclear as to whether IBTR is a marker of distant recurrence or a cause of it. Tuli *et al*. [36] have shown that following IBTR, the time to recurrence and the method of detection of IBTR (clinical *versus* mammographically) significantly influences development of distant disease. A study recently evaluating influence of IBTR on overall survival in five NSABP projects has significantly shown that those who do locally recur have a worse overall survival especially if receptor negative [37]. However the overall occurrence of IBTR at 10 years is 6.4% in node negative disease and 5.2% in those who receive adjuvant systemic treatment.

An emerging area of interest in these low risk groups is the use of accelerated partial breast irradiation. This has taken a number of forms including intraoperative radiotherapy with varying techniques and post-operative brachytherapy [38,39]. The early data are promising with regarding to feasibility with low rates of local recurrence [40,41,42,43]. However, guidelines regarding accelerated partial breast irradiation are cautionary given the lack of long-term effectiveness and safety data as compared to whole breast irradiation [44].

Current techniques are incapable of defining a large group of patients who do not need RT. One area of interest is the use of Magnetic Resonance Imaging in excluding multifocality and extensive intraductal component. A recent meta-analysis evaluating nineteen studies demonstrated that MRI detects additional disease in 16% of cases resulting in more extensive surgery by either larger wide local excisions or mastectomy [45]. The false positive rate remains an issue in MRI screening and MRI guided biopsy should be undertaken prior to decision regarding extent of surgery. However, MRI remains an exciting tool which may enable better definition of extent of disease and thus minimize risk of local recurrence.

## 4. Conclusions

The current evidence from randomized controlled trials highlights the importance of radiotherapy in local control after breast-conservation surgery. Radiotherapy decreases local recurrence across all groups of patients. The earlier trials involved heterogeneous patient groups, including tumours that were large, node positive, in all age groups and with variable resection margins. These trials have enabled identification of some low risk clinico-pathological features, which have resulted in the next generation of trials evaluating the need for adjuvant RT in highly selected patient groups. The PRIME II and BASO II may better define the value of radiotherapy in these groups.

The effect of radiotherapy on local recurrence in lower risk patients is smaller with and even smaller survival benefit. Defining ‘low risk’ remains a challenge. However, new tools with increased sensitivity for defining tumor extent, such as Magnetic Resonance Imaging may provide an opportunity to omit radiotherapy in truly low risk patients whilst developments in molecular markers from genetic or proteomic analysis of the cancer and the host may allow this low risk group to be eventually defined.
